# Consuming cassava as a staple food places children 2-5 years old at risk for inadequate protein intake, an observational study in Kenya and Nigeria

**DOI:** 10.1186/1475-2891-9-9

**Published:** 2010-02-26

**Authors:** Kevin Stephenson, Rachel Amthor, Sally Mallowa, Rhoda Nungo, Busie Maziya-Dixon, Simon Gichuki, Ada Mbanaso, Mark Manary

**Affiliations:** 1 Department of Pediatrics, St. Louis Children's Hospital, St. Louis, MO, USA; 2 Kenya Agricultural Research Insititute, Kakamega and Nairobi, Kenya; 3 International Institute of Tropical Agriculture, Ibadan, Nigeria; 4 National Root Crops Research Institute, Umudike, Abia State, Nigeria

## Abstract

**Background:**

Inadequate protein intake is known to be deleterious in animals. Using WHO consensus documents for human nutrient requirements, the protein:energy ratio (P:E) of an adequate diet is > 5%. Cassava has a very low protein content. This study tested the hypothesis that Nigerian and Kenyan children consuming cassava as their staple food are at greater risk for inadequate dietary protein intake than those children who consume less cassava.

**Methods:**

A 24 hour dietary recall was used to determine the food and nutrient intake of 656 Nigerian and 449 Kenyan children aged 2-5 years residing in areas where cassava is a staple food. Anthropometric measurements were conducted. Diets were scored for diversity using a 12 point score. Pearson's Correlation Coefficients were calculated to relate the fraction of dietary energy obtained from cassava with protein intake, P:E, and dietary diversity.

**Results:**

The fraction of dietary energy obtained from cassava was > 25% in 35% of Nigerian children and 89% of Kenyan children. The mean dietary diversity score was 4.0 in Nigerian children and 4.5 in Kenyan children, although the mean number of different foods consumed on the survey day in Nigeria was greater than Kenya, 7.0 compared to 4.6. 13% of Nigerian and 53% of Kenyan children surveyed had inadequate protein intake. The fraction of dietary energy derived from cassava was negatively correlated with protein intake, P:E, and dietary diversity. Height-for age z score was directly associated with protein intake and negatively associated with cassava consumption using regression modeling that controlled for energy and zinc intake.

**Conclusions:**

Inadequate protein intake was found in the diets of Nigerian and Kenyan children consuming cassava as a staple food. Inadequate dietary protein intake is associated with stunting in this population. Interventions to increase protein intake in this vulnerable population should be the focus of future work.

## Introduction

Adequate protein intake has been a concern and controversy in international nutrition community for the past 50 years [1,2]. Recently adequate protein intake has been defined by a World Health Organization (WHO) consensus review of the evidence [3]. While animal experiments have shown that isolated dietary protein deficiency results in wasting, stunting, weight loss, delay in worm expulsion, immune compromise and depressed levels of growth hormone [4-7], the clinical manifestations of isolated protein deficiency in human populations are not readily observed. Protein is often consumed in conjunction with zinc and energy [8]. All of these nutrients are essential for the normal function of almost all cellular and metabolic processes, and thus deficits in such nutrients have a myriad of generalized clinical effects. In sub-Saharan African 38% of children are stunted and 9% are wasted [9]. While the etiology of these anthropometric abnormalities is multi-factorial, some of these children subsist on diets with inadequate protein intake. The fraction of dietary energy derived from protein, the protein:energy ratio (P:E), is inversely correlated with stunting for many populations [10].

Cassava is the staple of more than 200 million individuals worldwide, most of whom live in Africa [11]. Cassava is drought tolerant and grows well in soils with modest nutrient composition. Worldwide, cassava production is approximately 225 million tons, an increase of 30% over the past 15 years [11]. Cassava, however, has the lowest P:E of any staple crop; the protein content among common cassava cultivars is typically only 1% [12]. Populations that consume large amounts of cassava may well be at risk for inadequate dietary protein intake.

This study tested the hypothesis that Nigerian and Kenyan children consuming cassava as their staple food are at greater risk for inadequate dietary protein intake than those children who consume less cassava.

## Methods

### Subjects

Subjects were healthy children aged 2-5 years living in areas where cassava is consumed as a staple food in southeast Nigeria and around Lake Victoria in Kenya. In Kenya, 450 healthy children were recruited for this study during April 2009. Children were excluded from participation if they had a chronic illness or disability, were breastfeeding, or were not permanent residents of the study area. If more than one child in a family was eligible, the younger was chosen to participate. This study was approved by the Human Research Protection Office of Washington University and by the Kenya Medical Research Institute.

In Nigeria, data collected in 2001-03 for the purposes of assessing the national nutritional status were used. Each state in Nigeria was assigned to an agro-ecological zone; all children surveyed who lived in the humid-forest zone, the area where cassava consumption is greatest, were included in this study. A child-mother pair was chosen from each randomly sampled house. If more than one child in a household was eligible, the youngest child was selected. Ethical clearance was granted to the survey from the Federal Ministry of Health through the Nutrition Division of the Ministry.

### Study design

A purposive sampling technique of Kenyan and Nigerian individuals. Children aged 2-5 years were chosen as the study population because they are a group in which significant statural growth occurs, they are no longer breast feeding, quantification of macronutrient intake by survey methods has been shown to be accurate, and they are a vulnerable group for whom dietary diversity is limited. In Kenya, children were recruited from 15 villages in the Kuria, Teso and Samia districts. From each village, a random sample of 30 children was chosen. A sample size of 450 was determined using estimates of iron intake data to achieve a precision of 10%. Considering each of the 3 districts to be independent and unrelated, a sample size of 144 per district was calculated.

In Nigeria, the states were selected based on their high reliance on cassava. The survey method has been described previously [13,14]. Briefly: within each state, a listing of all local government areas was obtained and these areas were then separated by degree of urbanization (rural, medium, and urban) [15,16]. The survey areas were selected with the goals of ensuring a range of rural/urban conditions. Local government areas were then divided into smaller demographic units. Three of these smaller demogrpahic units were randomly selected for the survey from a list. For each of these small units, 30 households were randomly chosen.

The primary outcomes were the Pearson Correlation Coefficients relating the fraction of dietary energy obtained from cassava and protein intake (g/kg/d) as well as the dietary P:E. Secondary outcomes were the correlation coefficients between the fraction of dietary energy obtained from cassava and measures of dietary diversity.

### Dietary recall + subject participation

In Kenya, a 24-hour recall method was used to estimate dietary intake [17,18]. Two days before the food recall, caregivers were asked to observe the quantities and types of food that their child consumed on a designated day. Each caregiver was given a graduated cup to help standardize quantities when cooking and feeding on that day. Caregivers were also asked to note any outside food source given to the children or any special circumstances (e.g. visiting family, special celebration or event). About 10% of children were surveyed twice using the same method to determine the variation in protein intake for the population. Caregivers also answered a questionnaire providing basic demographic and household information. Each child's weight was measured to the nearest 10 g and height was measured in triplicate to the nearest mm.

In Nigeria, a similar 24-hour recall method was used to estimate dietary intake [18]. Interviewers were provided with a food instruction booklet to collect descriptions of the foods consumed by respondents and their respective amounts. Each reported food description was compared to relevant food probes in the food instruction booklet. The respondents used measurement guides to approximate the amount of food eaten. Measurements consisted of: food models (e.g. model 1,2,3), prices (e.g. N5, N10, N30), volumes and measures (e.g. 10 ml, 1 cup, milk tin), and sizes (small, medium, large). The questionnaire also included information about household composition; socioeconomic status of the household; trends in food availability/frequency of food consumption, and household food expenditures. All interviews were conducted in person with the child's caretaker, usually his/her mother. Samples of Nigerian food prepared and eaten by household members were subjected to compositional analysis where possible [14].

The field workers conducting the 24 hour dietary recalls completed a 1 week training course prior to beginning data collection, including validation/standardization of their survey techniques [14,18].

### Data analysis

Each subject was assigned a study number unlinked to identifiers. Data were entered into a Microsoft Excel spreadsheet. Protein and energy intake for each Kenyan subject was calculated using two East African food composition tables [19,20]. Dietary protein and energy information was also obtained from the USDA databases for the few foods not included in the East African database [21]. For the Nigerian subjects, original data collection sheets were not available, but instead only the form of an aggregate spreadsheet without identifiers because this study made secondary use of the original survey data.

Dietary diversity was scored by 3 methods. First, all foods were categorized into one of 12 general types, such as cereals, fish, sugar, and fats, and children were given a 12 point score [22]. The second method defined whether animal-source foods were consumed on the survey day, and the final method used a numerical count of the number of different foods eaten on the survey day [23].

The WHO reference value for adequate protein intake for children 2-5 aged years, 1.1 g/kg/d, was used as the standard for adequate intake [3]. This reference value was determined using multiple studies of nitrogen balance at varying intakes for children in addition to the factorial approach [3]. The adequate P:E was also calculated using the WHO reference value for energy requirements for a population of children 2-5 years of age and the WHO reference value for protein intake. A P:E of 5.0% for males and 5.3% for females was deemed as adequate [24]. Anthropometric indices were calculated using the World Health Organization 2005 growth standards [25]. Pearson Correlation coefficients were determined between the fraction of energy obtained from cassava and protein intake(g protein/kg) as well as P:E (SPSS 17.0, Chicago).

Multivariate stepwise linear regression analysis was conducted to determine if height-for-age z score was associated with dietary protein intake and/or cassava intake. Covariates included in the model were sex, age, country of residence, use of clean water, as well as energy, zinc, iron, and Vitamin A intake.

Protein intake in the study population was also estimated using a protein content of cassava that was 4 and 8 times the amount typically found, keeping all other dietary intake parameters the same, to determine the effect a protein fortified cassava might have on protein intake.

## Results

Data from 449 Kenyan and 656 Nigerian children were included in the study (Table 1). For the Kenyan diet, cassava comprised 59% of a subject's daily energy intake, while in Nigeria, maize comprised 22% of total energy intake and cassava 15% (Table 2).

**Table 1:** Demographic, anthropometric and socioeconomic profile of study populations

	Nigeria n = 656	Kenya n = 449
Sex, male	322 (49%)	220 (49%)
Age (mo)	38 ± 10	40 ± 10
Weight-for-height z-score	0.0 ± 1.2	-0.2 ± 0.9
Height-for-age z-score	-1.5 ± 1.8	-1.6 ± 1.3
Weight-for-age z-score	-0.8 ± 1.3	-1.0 ± 1.0
Weight (kg)	13.1 ± 2.6	13.2 ± 2.1
Stunted, height-for-age < -2 z score	236 (36%)	162 (36%)
Occupation of head of household		
Farming	278 (32%)	330 (75%)
Trading	136 (16%)	15 (3%)
Civil servant	118 (14%)	5 (1%)
Artisan	175 (20%)	60 (14%)
Fisherman	11 (1%)	1 (-)
Other	149 (17%)	29 (7%)
Home is electrified	452 (52%)	0
Uses a source of clean water (city water, borehole)	298 (34%)	198 (44%)

(Data expressed as n (%) or mean ± standard deviation)

**Table 2:** Sources of energy in the diets of cassava consuming children

Food	Nigeria n = 656	Kenya n = 449
Cassava	15%	59%
Maize	22%	7%
Rice	14%	1%
Sorghum	1%	10%
Wheat	8%	1%
Animal source foods	3%	7%
Legumes	9%	3%
Fruits	4%	3%
Green leafy vegetables	10%	4%
Yams	11%	0%
Banana	2%	1%
Sweet Potato	0%	3%

(Data expressed as fraction of daily dietary energy)

Inadequate protein intake was found in 13% of the Nigerian children studied and in 53% of Kenyan children (Table 3). The diets of both populations had a mean diversity score, < 4.5 (Table 3). The fraction of energy in the diet provided by cassava was inversely correlated with protein intake, with P:E, with dietary diversity, with the number of different foods consumed, and with the consumption of animal-source foods (Table 4).

**Table 3:** Diet survey of cassava consuming children

	Nigeria n = 656	Kenya n = 449
Protein Intake		
Total protein intake (g/kg)	2.5 ± 1.2	1.2 ± 0.9
Dietary protein: energy ratio	9.7 ± 3.8	8.4 ± 3.8
Children with protein intake ≥ 1.1 g/kg*	569 (87%)	212 (47%)
Children with protein-energy ratio ≥ 5.3%	596 (91%)	245 (55%)

Energy, zinc and iron intake		
Energy intake (kcal/kg)	84 ± 10	76 ± 8
Zinc intake (mg)	3.1 ± 1.8	3.3 ± 2.2
Iron intake (mg)	9.1 ± 5.8	6.0 ± 4.1

Cassava consumption		
> 50% of dietary energy from cassava	74 (11%)	295 (66%)
25-50% of dietary energy from cassava	156 (24%)	103 (23%)
15-25% of dietary energy from cassava	102 (15%)	17 (4%)
5-15% of dietary energy from cassava	130 (20%)	15 (3%)
<5% of dietary energy from cassava	194 (30%)	19 (4%)

Dietary Diversity		
Household dietary diversity score (0-12)**	4.0 ± 1.4	4.5 ± 1.3
Number of different food items consumed	7.0 ± 2.7	4.6 ± 1.2
Consumed an animal-source food	271 (41%)	319 (71%)

Data expressed as mean ± standard deviation or n (%)

*Chosen to an adequate level of protein intake on the basis of WHO/FAO/UNU consultation [3]

**FANTA Household Dietary Diversity Score [22]

**Table 4:** Pearson's Correlation Coefficients between dietary cassava consumption and protein intake/diet quality

	Nigeria n = 656	Kenya n = 449
Fraction energy from cassava and P:E	-0.30*	-0.45*
Fraction energy from cassava and protein intake	-0.18*	-0.42*
Fraction energy from cassava and 12 point dietary diversity score	-0.28*	-0.33*
Fraction energy from cassava and number of different foods consumed	-0.29*	-0.26*
Fraction of energy from cassava and consumption of animal-source foods	-0.22*	-0.15*

* All coefficients significant at a *P *< 0.01

Fifty-four children, all in Kenya, had two dietary assessments on separate days to test variability in protein intake. The first survey found an average protein intake of 16 ± 7 g while the second survey found 14 ± 7 g, a variation of 7% in the population's consumption.

Linear regression modeling found that that protein intake was directly related to height-for-age z score and cassava intake was negatively related with height-for-age z score (r = 0.25, *P *< 0.001 for the model, standardized coefficients were 0.20 and -0.11 respectively, *P *< 0.001 for both coefficients); energy, zinc and iron intake were all significant covariates in the model, with energy being the most important of the covariates. Age and sex were significant covariates in the model, but consumption of clean water was not significant.

If the P:E was increased 4-fold, the fraction of Kenyan children with inadequate protein intake would decrease to 32%, while the fraction of Nigerian children with inadequate intake would decrease to 10%. If the P:E was increased 8-fold, 13% of Kenyan children would have had inadequate protein intake and 8% of Nigerian children would have had inadequate intake.

## Discussion

This study found that cassava intake is inversely correlated with protein intake and diet quality in Nigeria and Kenya. Those individuals who consume cassava as a staple do not compensate for the very low P:E of cassava by including sufficient amounts of protein-rich foods such as legumes and fish in the diet.

A limitation of this study is the uncertainty associated with a single 24 hour recall to determine dietary intake, as dietary intake is known to vary substantially day-to-day [17,18]. A series of consecutive recalls is more accurate than a single one for determination of an individual's dietary intake. The value of our single dietary recall was assessed by repeating the recall in 54 children, and a 7% variation in protein intake was found for this group of children. However, it is established that when considering a population, a single dietary recall provides accurate estimate of the nutrient intake and deficits of the population as a whole [18,26]. Another limitation is that our study population was predominantly of rural Africans, who, for the most part, were subsistence farmers, thus our findings should not be extended to laborers who primarily purchase food, nor to urban dwellers. Thirdly, this assessment was restricted to 2-5 year old children and therefore it might not be applicable to other age groups who obtain more of their food prepared outside the home. The analyses exploring the relationship between stunting and protein intake is limited in that this study only provides information at a single point in time, stunting is a dynamic process that certainly is influenced not only by diet, but the frequency of acute infections.

The typical protein content of cassava is about 1.3 g/100 g. A serving of cassava that constitutes 25% of an average energy intake of a 2-5 year old provides only 1.1 g protein, about 8% of the estimated protein requirement. The Protein Digestibility Corrected Amino Acid Score (PDCAAS) for cassava is 57, one of the lowest on any staple foods, with lysine and leucine being the limiting amino acids [24,27]. These facts suggest that it is difficult for children to get adequate dietary protein from cassava, and children that do receive a significant fraction of their dietary protein from cassava are at risk for inadequate essential amino acid intake.

The protein intake of the Nigerian population studied was greater than that of the Kenyan population. This was achieved by incorporating more low cost cereals in the diets, such as maize, rice and sorghum, rather than foods nutritionists traditionally identify as good sources of dietary protein, such as fish or legumes.

Dietary diversity has been shown to be linked to household food security, child growth, and diet quality. Diets were found to be less diverse as reliance on cassava as a staple food increased. This association identifies cassava consumers as a vulnerable population that may benefit from interventions to improve nutrition.

Our finding that dietary protein intake is a predictor of height is consistent with previous results in other settings. In China, height correlated with dietary protein intake, when controlled for dietary energy intake, socio-economic status and income [10]. Another study compared adult heights by country with dietary energy and protein intake, income, ethnic group and socio-economic status, and only dietary protein intake was associated with height [10]. A study from Peru found that animal source dietary protein was strongly associated with height and weight, but not with dietary energy [28]. Studies from Bangladesh and the Philippines show that dietary protein intake, but not dietary energy intake, is associated with child growth [29,30]. Certainly these associations between growth and protein intake do not prove a causal relationship, and these previous studies did not account for the micronutrient content of the diets as confounding factors. But the cumulative evidence from observational human studies and animal intervention studies strongly supports the notion that if the P:E of the diet falls below a certain level during the time when statural growth occurs, the result is stunting. The WHO estimates of adequate protein intake and energy requirements suggest that a P:E < 5% in humans is detrimental. While it is rare to see such low P:Es, our data indicate that such diets are not uncommon among populations that consume cassava as a staple food.

Interventions aimed at increasing dietary protein intake to improve growth and reduce morbidity in children include nutritional education and counseling and supplemental feeding programs. Typically studies have found very modest correlations between nutrition knowledge and dietary behavior [31-33]. A study in infants analyzed the effect of maternal nutritional counseling over 12 months and found no increase in weight or height gain and no decrease in morbidity [34]. Supplemental feeding programs usually involve blended flours, which are combinations of cereals, legumes, and other foods to provide high quality protein derived from plants, given over a period of several weeks to a vulnerable population [35]. Although these programs are extensive, expensive, and have been used over many decades, there is no evidence that supplemental feeding reduces or reverses stunting [36]. Given the meager effectiveness of education and supplemental feeding in the past, it seems unlikely that they would be helpful to very poor populations that consume cassava as a staple food in the future.

The protein content of existing cassava cultivars varies between 1-4% [37], with a typical value of 1%. Hence breeding of the most protein-rich cassavas with African germplasm for consumption as a staple will not result in cassava hybrids that will ameliorate dietary protein deficiency. A novel research initiative, BioCassava Plus, aims to achieve increased protein content through genetic engineering of local cassava varieties of cassava consuming populations. If the P:E of cassava were increased 4-8 fold using genetic enhancement of cassava, the fraction of the population with inadequate protein intake might be reduced to half of that found in this study.

## Conclusion

This study found that cassava intake is inversely correlated with protein intake and diet quality in 2-5 year old children in Nigeria and Kenya. Among Kenyan children, 53% had inadequate protein intake and 13% of Nigerian children had inadequate protein intake. This study indicates that populations that consume cassava as a staple are at risk for inadequate protein intake. Further work is needed to increase the dietary protein intake of this vulnerable population.

## Competing interests

The authors declare that they have no competing interests.

## Authors' contributions

KS, RA, BMD, SG, AM and MM designed the study. RA, SM, RN, BMD, and MM collected the data. KS, RA, RN, BMD and MM analyzed the data. KS wrote the first draft of the manuscript. All authors edited and approved the final manuscript.

## References

[B1] Scrimshaw NS Behar M Protein malnutrition in young children Science 1961 133 2039 2047 10.1126/science.133.3470.2039 13749432

[B2] McLaren S The great protein fiasco Lancet 1974 2 93 96 10.1016/S0140-6736(74)91649-3 4137270

[B3] Protein amino acid requirements in human nutrition, report of joint WHO/FAO/UNU expert consultation 2007 WHO Technical Report Series: 935 WHO, Geneva

[B4] Atinmo T Baldijao C Pond WG Barnes RH Prenatal and Postnatal Protein Malnutrition in Pigs: Effects on Growth Rate, Serum Protein and Albumin J Anim Sci 1976 43 606 612 824264 10.2527/jas1976.433606x

[B5] Cummins A Bolin T Duncombe V Davis A The effect of methionine and protein deficiency in delaying expulsion of *Nippostrongylus brasiliensis *in the rat Am J Clin Nutr 1986 44 857 862 3788833 10.1093/ajcn/44.6.857

[B6] Tetzlaff CL Carlomagno MA McMurray DN Reduced dietary protein content suppresses infection with *Babesia microti* J Mel Micro Immunol 1988 77 305 315 10.1007/BF02389902 3216813

[B7] Tirapegui J Baldi M Ribeiro S Effect of protein deficiency on plasma insulin-like growth factor-I (IGF-I) level on protein and proteoglycan synthesis rates in skeletal muscle and bone Nutr Res 1996 16 869 879 10.1016/0271-5317(96)00080-2

[B8] Golden M Oedematous Malnutrition Brit Med Bull 1998 54 433 444 9830208 10.1093/oxfordjournals.bmb.a011699

[B9] Unicef State of the World's Children 2009 New York 2009

[B10] Jamison DT Leslie J Musgrove P Malnutrition and dietary protein: Evidence from China and from international comparisons Food Nutr Bull 2003 24 2 FAO 11 10.1177/156482650302400201 12891820

[B11] Faostat data http://faostat.fao.org/site/567/DesktopDefault.aspx?PageID=567#ancor

[B12] Stupak M Vanderschuren H Gruissem W Zhang P Biotechnological approaches to cassava protein improvement Trends Food Sci Technol 2006 17 634 641 10.1016/j.tifs.2006.06.004

[B13] Maziya-Dixon BB Akinyele IO Sanusi RA Oguntona TE Nokoe SK Harris EW Vitamin A deficiency is prevalent in children less than 5 y of age in Nigeria J Nutr 2006 136 2255 2261 16857850 10.1093/jn/136.8.2255

[B14] Maziya-Dixon B Nokoe S Manyong V Harris E Akinyele IO Oguntona EB Sanusi RA Report on Survey Design and Operation: Nigeria Food Consumption and Nutrition Survey 2001-2003 2006 International Institute of Tropical Agriculture, Ibadan, Nigeria

[B15] Okali D Okpara E Olawoye J Rural-urban interactions and livelihood strategies series. The case of Abia and its region, southern Nigeria. Working paper 4 2001 London, UK: International Institute for Environmental and Development (IIED)

[B16] Tacoli C Changing rural-urban interactions in sub-Saharan Africa and their impact on livelihoods: a summary 2002 London, UK: International Institute for Environment and Development

[B17] Karvetti RL Knuts LR Validity of the 24-hour Dietary Recall J Am Diet Ass 1985 85 1437 1442 4056262

[B18] Gibson RS Ferguson EL An interactive 24-hour recall for assessing the adequacy of iron and zinc intakes in developing countries Harvest Plus technical monograph series 2008 Harvest Plus UN University

[B19] Lukmanji Z Hertzmark E Mlingi N Assey V Ndossi G Fawzi W Tanzania Food Composition Tables 2008 Muhumbili School of Health and Allied Sciences, Tanzania http://www.hsph.harvard.edu/nutritionsource/files/tanzania-food-composition-tables.pdf

[B20] The WorldFood Dietary Assessment System (NFOOD) (1997) WFOOD, Version 2.0 Berkeley, CA: University of California, Berkeley [computer program]

[B21] USDA National nutrient database for standard reference http://www.ars.usda.gov/main/site_main.htm?modecode=12-35-45-00

[B22] Swindale A Bilinsky P Household dietary diversity score (HHDS) for measurement of household food access: indicator guide Food and Nutrition Technical Assistance Project, Washington 2006

[B23] Ruel M Is dietary diversity an indicator of food security of dietary quality? A review of measurement issues and research needs IFPRI FCND Discussion Paper 140, Washington 2002 10.1177/156482650302400210 12891828

[B24] Milward DJ Jackson AA Protein/energy ratios of current diets in developed and developing countries compared with a safe protein/energy ratio: implications for recommended protein and amino acid intakes Public Health Nutr 2003 7 387 405 10.1079/PHN2003545 15153271

[B25] WHO Anthro 2005 Beta http://www.who.int/childgrowth/software/en/

[B26] Thompson FE Byers T Dietary Assessment Resource Manual J Nutr 1994 124 2245 2317 10.1093/jn/124.suppl_11.2245s 7965210

[B27] Ngudi DD Kuo UH Lambein F Food safety and amino acid balance in processed cassava "cossettes" J Agric Food Chem 2002 50 3042 9 10.1021/jf011441k 11982439

[B28] Graham GG Creed HM MacLean WC Kallman CH Rabold J Melitis ED Determinants of growth among poor children:nutrient-intake achieved growth relationships Am J Clin Nutr 1981 34 539 54 7223704 10.1093/ajcn/34.4.539

[B29] Becker S Black RS Brown KH Relative effects of diarrhea, fever and dietary energy intake on weight gain in rural Banglideshi children Am J Clin Nutr 1991 53 1499 1503 2035479 10.1093/ajcn/53.6.1499

[B30] Bhargava A Modelling the health of Filipino children J Statist Soc Ser A 1994 157 417 432 10.2307/2983528

[B31] Rasanen M Niinikoski H Keskinen S Helenius H Talvia S Ro¨nnemaa T Viikari J Simell O Parental nutrition knowledge and nutrient intake in an atherosclerosis prevention project: the impact of child-targeted nutrition counseling Appetite 2003 41 69 77 10.1016/S0195-6663(03)00046-1 12880623

[B32] Axelson ML Federline TL Brinberg D A meta-analysis of food and nutrition related research J Nutr Educ 1985 17 51 54

[B33] Stafleu A Van Staveren WA De Graaf C Burema J Nutritional knowledge and attitudes towards high-fat foods and low fat alternatives in three generations of women Eur J Clin Nutr 1996 50 33 41 8617189

[B34] Bhandari N Bahl R Nayyar B Khokhar P Rohde JE Food Supplementation with Encouragement to Feed It to Infants from 4 to 12 Months of Age Has a Small Impact on Weight Gain J Nutr 2001 131 1946 1951 11435512 10.1093/jn/131.7.1946

[B35] Dewey KG Brown KH Update on technical issues concerning complementary feeding of young children in developing countries and implications for intervention programs Food Nutr Bull 2003 24 5 28 12664525 10.1177/156482650302400102

[B36] Navarro-Colorado C Mason F Shoham J Measuring effectiveness of supplementary feeding programmes in emergencies Network Paper No. 63 2008 Humanitarian Practice Network, London

[B37] Ceballos H Sanchez T Chavez AL Iglesias C Debouck D Mafla G Tohme J Variation in crude protein content in cassava (Manihot esculenta Crantz) roots J Food Compost Anal 2006 19 589 593 10.1016/j.jfca.2005.11.001

